# Investigating the Impact of Hardness on Dielectric Breakdown Characteristics of Polyurethane

**DOI:** 10.1021/acsomega.4c00509

**Published:** 2024-06-03

**Authors:** Abdul Samad, Wah Hoon Siew, Martin Given, John Liggat, Igor Timoshkin

**Affiliations:** † Department of Electronic and Electrical Engineering, University of Strathclyde, Glasgow G1 1XQ, U.K.; ‡ Department of Pure and Applied Chemistry University of Strathclyde, Glasgow G1 1XQ, U.K.

## Abstract

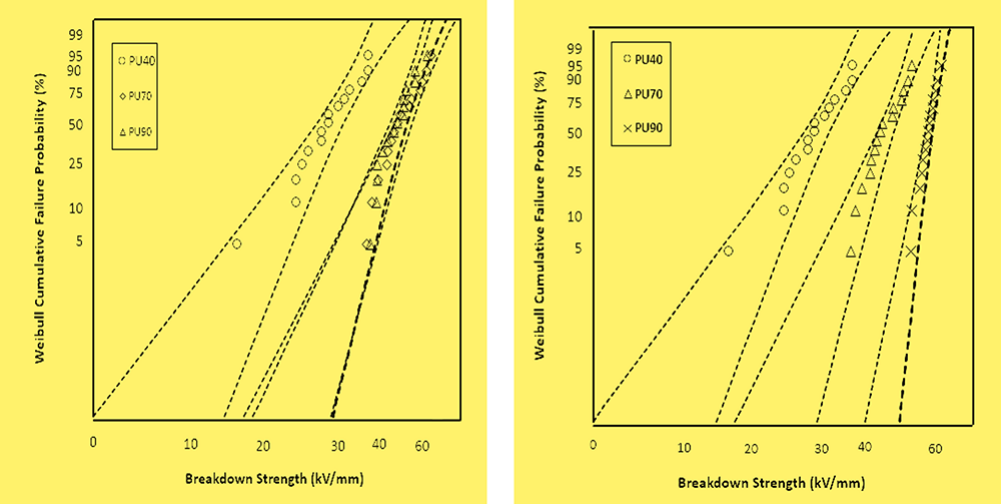

Polymeric materials play a vital role in high-voltage insulation, but their insulating properties can deteriorate over time, leading to insulation failures. The presence of voids resulting from manufacturing defects or external stresses can create a highly divergent field, further contributing to this issue. However, certain polymers, such as polyurethane (PU), possess self-healing properties that enable them to repair these voids and restore a uniform electric field distribution, thereby ensuring the reliability of the insulation. Surprisingly, the potential of PU as an insulating material in high-voltage applications remains unexplored. However, the self-healing capability of PU decreases with an increase in the hardness of the material. Therefore, in this study, the dielectric breakdown properties of PU with different levels of hardness, rated on the Shore scale as 40° (soft), 70° (medium), and 90° (hard), were investigated. The AC and DC dielectric breakdown characteristics of these PU variants and dielectric spectra were examined. Additionally, the study explores the relationship between the dielectric properties and the hardness of the material. Our findings revealed that the dielectric breakdown strength of PU increases as the material’s hardness is increased under both AC and DC electric stress. However, this may come at the cost of reduced self-healing capabilities of PU. Therefore, there is a need to balance the hardness of the material with its ability to recover from breakdown events. The findings from this study can be useful for researchers and engineers, as they offer valuable insights into the dielectric properties of PU at various hardness levels.

## Introduction

The electricity demand has increased in recent years, leading to the expansion of electric power networks across the globe. As a result, innovative approaches are required to meet the public’s energy requirements while ensuring the reliability of power transmission and distribution. One critical aspect of this process is the choice of the cable insulation material. The insulation material plays a vital role in ensuring that power utilities operate safely and efficiently. The move from traditional ceramic and oil-paper insulation to polymeric materials has been a remarkable change in high-voltage insulation. Because of easy processing, high resistance to degradation, low cost, and high dielectric strength, crossed-link polyethylene (XLPE) is widely used as an insulating material in high-voltage applications.^1,2^ XLPE is degraded by electric stress and water.^3,4^ Initially, it was believed that water from residual moisture, environment, and surroundings penetrates the polymer and forms a water tree which, on drying, leaves behind its traces.^5,6^ These channels or tubules provide the conducting path to the electric charges. Over time, this can lead to the initiation of a partial discharge (PD) or the formation of electrical trees. Usually, water trees do not cause an insulation breakdown, but these can create PD or electrical trees, which are recognized as the primary factors contributing to insulation failure.^7^ To overcome this problem, researchers tried to improve the manufacturing technology by adding water tree retardant material and developing semiconducting materials with higher purity.^8−10^ These methods helped by delaying the formation of electrical trees but could not eliminate the whole problem. Later, it was found that not only the traces of water trees can initiate the formation of electrical trees but the presence of protrusion or roughness of the surface of the insulating polymers can also initiate the formation of electrical trees.^11−13^ Moreover, at the interphase of different materials during the manufacturing of power cables, the formation of voids or cavities cannot be entirely avoided. Under high electric stress, these voids can initiate the PD or electrical trees, which propagates through the insulation and causes the insulation breakdown.^14,15^

**Figure 1:**
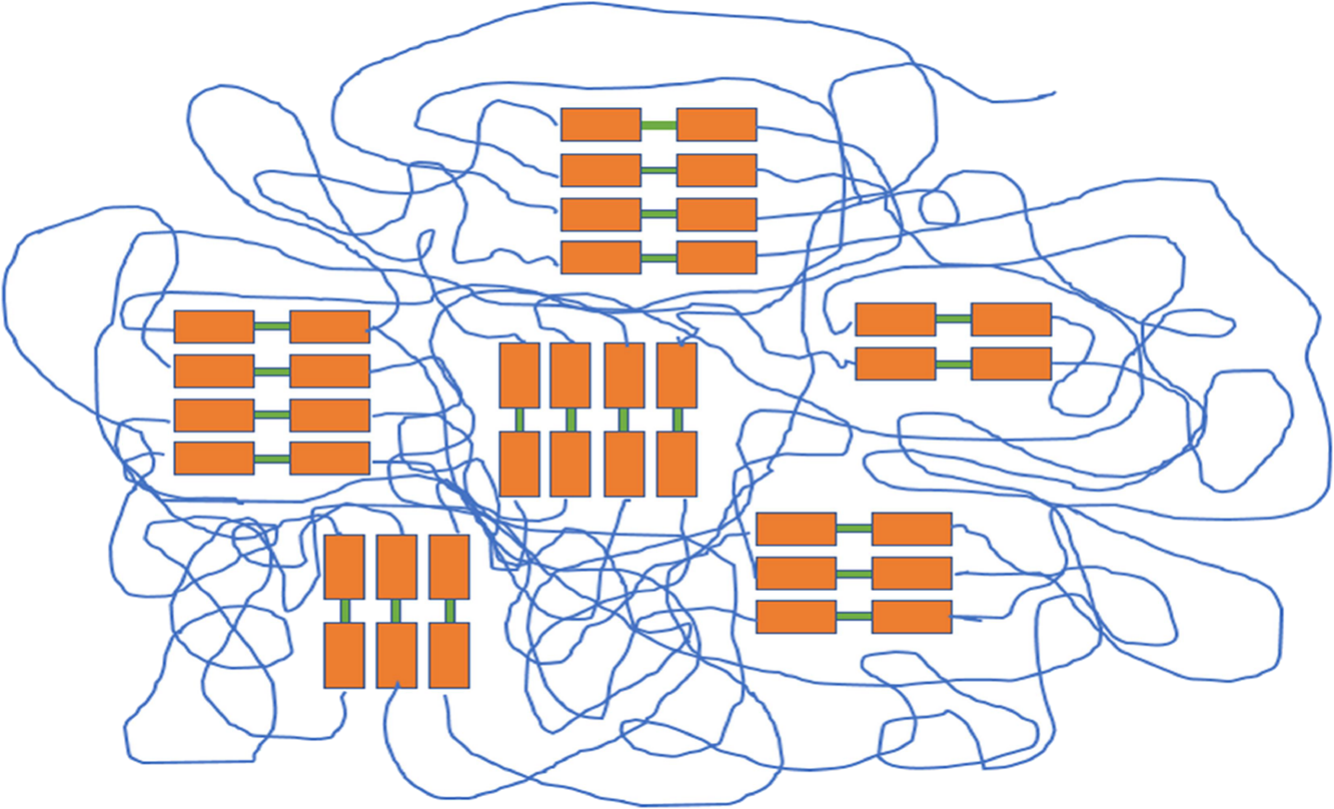
Structure of PU. Thin blue lines represent soft segments, orange rectangles represent hard segments, and green rectangles represent chain extenders.

Exploring new insulation materials with good dielectric characteristics and robust mechanical strength is crucial to ensuring reliability and continuous electric supply. These materials should also be capable of effectively addressing the defects that may arise due to manufacturing faults, the presence of water tree traces, or mechanical stresses. Polyurethane (PU) is a well-established material studied for its inherent self-healing properties. It is renowned for its ability to recover effectively from surface dents due to its high elasticity. PUs are composed of urethane repeating units. The synthesis of PUs involves polymerizing polyisocyanates and macropolyols and the inclusion of chain extenders.^16−18^ The macropolyols, such as polyether diols or polyester diols, are the soft segments. In contrast, the isocyanates (aromatic or aliphatic) and chain extenders (small diols or diamines) are known as the hard segment. Figure 1 shows the structures of the PUs. The hard phase in PUs contributes to their mechanical strength. Meanwhile, the flexible polymer chains in PUs provide them with the desired characteristics of flexibility and elasticity. By carefully selecting the appropriate raw materials and adjusting the relative ratios of the hard and soft segments, the phase-separated structure can be easily modified, leading to variations in the properties of PU. However, it is important to note that the self-healing capability of PU is limited when it comes to healing scratch damage areas, primarily due to the absence of strong specific interactions between its polymer chain. Additionally, PU, like many other self-healing materials, faces a trade-off between mechanical strength and self-healing capacity, which can result in compromised restoration of mechanical properties after damage occurs.^17,19^

However, a significant challenge lies in understanding the dielectric properties of PU. This study aimed to investigate the dielectric characteristics of PU with different levels of hardness. It also aimed to analyze the intricate balance between dielectric properties and hardness of the PU materials, as this interplay is closely linked to the self-healing capability of PU.

## Experimental Section

### Materials

One-meter-long PU rods with varying hardness levels were obtained from the PAR Group Ltd. to conduct the study. The PU rods were categorized into three distinct categories based on the Shore scale, namely soft (40°A), medium (70°A), and hard (90°A). This classification enables a comprehensive analysis of the effects of hardness on the dielectric properties and performance of PU materials with varying hardness under electric stress.

### Preparation of Samples

To conduct the study, small pieces weighing 300 mg were cut from each type of PU rod and were melted to make the thin films. The melting process was conducted using a 15 ton manual hydraulic press machine from Graseby Specac, employing a temperature of 200 °C. A thin sheet of aluminum with a thickness of 300 μm with a circular hole of diameter 45 mm was used to act as a mold. A small piece of poly(tetrafluoroethylene) (PTFE) film, measuring 50 μm in thickness and 70 mm in diameter, was placed on the lower plate of the hot press. The mold was then carefully positioned on top of the PTFE film, ensuring that the hole of the mold was covered by the PTFE film.

The small pieces of polymeric rods were placed at the center of the hole on top of the smooth PTFE film, and an additional piece of smooth PTFE film was added on top of the mold to cover the polymeric pieces, preventing direct contact with the hot plates and allowing for uniform heating from both the top and bottom plates of the hydraulic press. The use of the PTFE film was advantageous due to its higher melting point and its ability to enable the melted polymer to cool and form a thin sheet on its surface. This thin polymeric film deposited on the PTFE film could be easily isolated, as it did not stick to the film after cooling, making it a highly efficient and successful method for the preparation of thin smooth films for further analysis. Previous studies have shown that surface roughness can affect the observed dielectric strength of synthesized PU films, with a lower thickness leading to increased roughness and discrepancies in observed dielectric strength, particularly for films with thickness below 10 μm. However, for films with a smooth surface and thickness higher than 10 μm, the effects of surface roughness on dielectric strength measurement are negligible.^20,21^

The samples were carefully positioned between the hot plates for 15 min while a 5 ton load was applied. The load was released and reapplied multiple times to eliminate trapped air within the samples. Afterward, the melted PU was allowed to cool under the hydraulic press until the temperature dropped to 75 °C. Following this, the samples were gently removed from the hydraulic press alongside the hot plates and allowed to cool at room temperature for 5 min. This process resulted in smooth thin films of PU. Subsequently, the thickness of these thin samples was measured using a micrometer (Mitutoyo, 0–25 mm, 0.01 mm), resulting in thin films with an average thickness of 260 μm. To simplify and enhance clarity throughout the study, the PU samples with Shore scale hardness ratings of 40°A, 70°A, and 90°A will be referred to as “PU40”, “PU70”, and “PU90”, respectively. These designated labels will be consistently used to facilitate easy reference and comparative analysis of the different PU materials.

## Characterization

### Structure of Polymers

Before the small pieces of each type of PU were subjected to the melting process in the hot press, Fourier transform infrared (FTIR) spectroscopy was employed to examine the composition of the samples. The small pieces of each PU variant were evaluated with a Nicolet iS5 FTIR spectrometer with a Specac GoldenGate ATR accessory, to obtain the FTIR spectra. Data was collected by averaging 64 scans from 500 to 4000 cm^–1^ at a resolution of 4 cm^–1^. The resulting data were recorded for analysis. Furthermore, the process was repeated on the thin sheets synthesized from each material using the hot press. This additional investigation aimed to gain valuable insights into the potential influence of the molding process and ascertain the presence of any noticeable physical or chemical changes in the material structure resulting from the molding process.

### Differential Scanning Calorimetry

The thermal behavior of the polymers was characterized using differential scanning calorimetry (DSC). The DSC measurement was conducted by using a DSC Q1000 TA Instruments. The instrument was equilibrated at a low temperature of −40 °C to ensure a stable baseline. This is essential as it helps to establish a consistent DSC measurement starting point equilibration; the temperature is then ramped up at a rate of 10 °C per minute,^22^ reaching a maximum temperature of 300 °C, while N_2_ was used as a purge gas with a flow rate of 40 mL min^–1^. However, it is important to note that different polymers may require different heating rates; nevertheless, previous research has indicated that employing a ramping rate of 10 °C per minute effectively captures all the significant thermal events in PU.^22−25^ This controlled heating process allows the sample to undergo physical transitions such as the glass transition, hard-block melting, and chemical changes such as decomposition.

### Dielectric Spectra and DC Conductivity

The dielectric response of the prepared samples was evaluated by using dielectric spectroscopy across a range of frequencies. The samples, with a diameter of 40 mm and an average thickness of 260 μm, were positioned between stainless steel parallel plate electrodes. The current sensing electrode had a guard ring and an effective diameter of 35 mm, eliminating the effects of fringing fields and leakage currents. Ten samples of each type of PU were used to measure the dielectric properties, and the variation in output data was further analyzed to calculate the percentage variation in dielectric properties and subsequent edge effects.

An LCR meter (ET4510, East Tester) operating in AC mode was employed for the measurements, covering a frequency range of 100 Hz to 100 kHz with a signal amplitude of 1 V.

In addition to the dielectric response, the DC conductivity of each PU variant was assessed using the same samples and electrode setup. A Keithley 617 programmable electrometer was utilized to apply a range of DC voltages from 10 to 100 V, simultaneously recording the corresponding output current. Due to the presence of charging transients when the voltage was applied, it was necessary to allow a period of time for the current to stabilize to its DC value for each voltage input. To ensure the reliability and accuracy of the output DC current values, an initial approach involved recording DC current values at 5, 10, and 15 min. It was noted that there was no observable change in magnitude between the DC current values recorded at 10 and 15 min. As a result, a 10 min time delay was implemented before recording the current value.

### Dielectric Breakdown Testing of PU Variants

Alternating Current (AC) and Direct Current (DC) breakdown tests were conducted on the synthesized thin films of PU40, PU70, and PU90. These tests involved placing the thin disc film samples (40 mm diameter, 260 ± 10 μm thickness) in a spherical-plain electrode system, wherein a 3 mm diameter copper ball bearing served as the high-voltage electrode, and a plain electrode acted as the grounding electrode.

**Figure 2:**
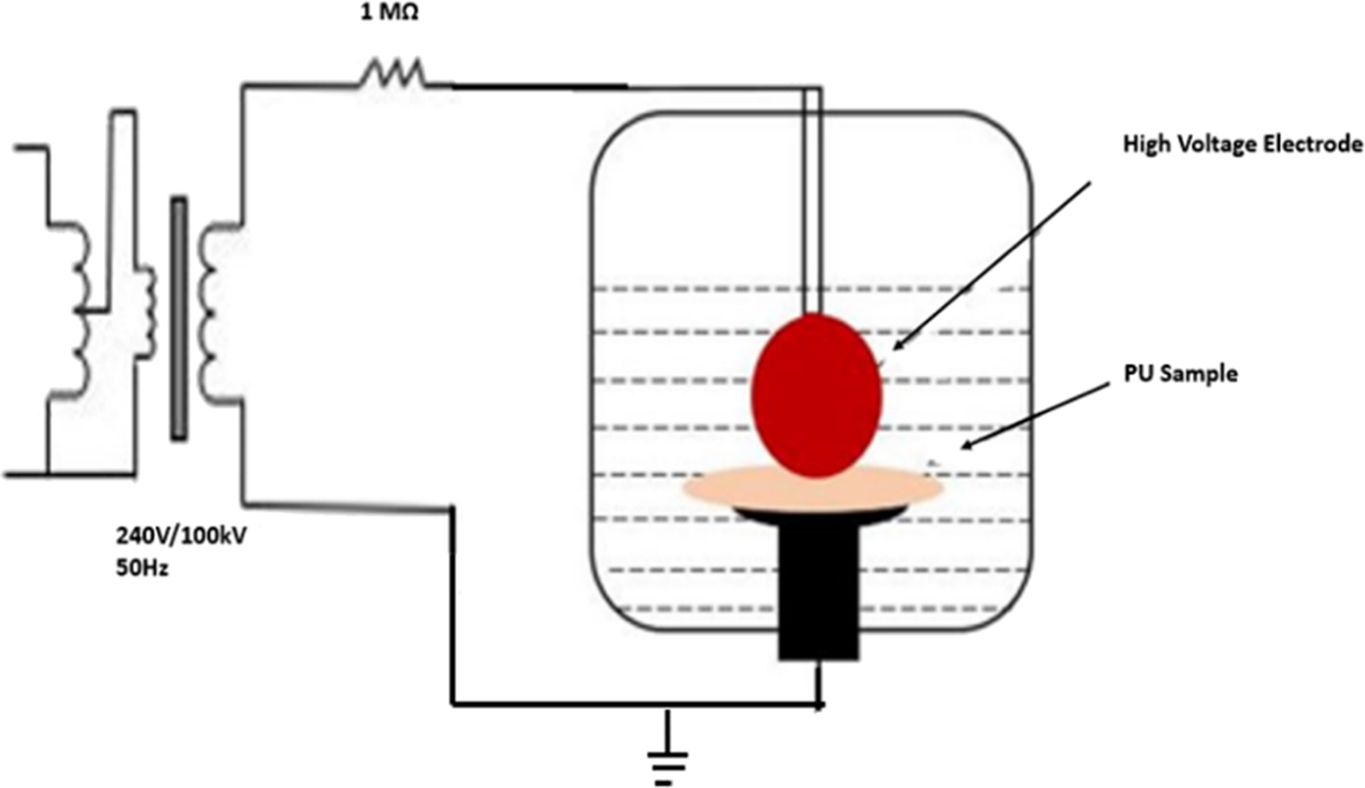
Electrical breakdown test setup.

To prevent surface flashover, the electrodes and the samples were immersed in transformer oil (Synthetic Ester MIDEL 7131). The plain electrode was fixed a little above the bottom of the test cell in transformer oil, and the sample was placed on the plain electrode. The spherical electrode was mounted on the end of a rod, which passed through a mounting in the lid of the test cell. The position of the spherical electrode was then adjusted to ensure that it was in contact with the sample without subjecting it to pressure, and the mounting was used to lock the position of the rod. After every breakdown test, the lid of the test cell was removed and then placed again as shown in Figure 2 . This ensured consistent pressure on all samples under the test. The tests followed the ASTM D149 and IEC 60234 standards, employing a short-time test technique. An AC voltage with a step voltage of 1 kV every 20 s and a DC voltage having positive polarity with a step voltage of 2 kV every 20 s were applied until the sample punctured. Each type of material underwent 15 breakdown tests, while each sample underwent only three tests at different positions to prevent any potential flashover at the punctured sites. The breakdown voltage was then divided by the thickness of the thin film to determine the breakdown field. It is important to note that the variation in the thickness of the PU films can affect the measurement of the breakdown voltage, thereby impacting the accuracy of the calculated breakdown field strength. Therefore, to accurately analyze the measured breakdown voltage and the variation in the measured breakdown strength, the resulting breakdown data were analyzed statistically using the two-parameter Weibull distribution, represented by eq 1.
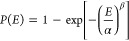
1where *P*(*E*) is the cumulative probability of failure at *E*, *E* is the experimental breakdown strength, α is the scale parameter representing the breakdown strength at the cumulative failure probability of 63.2%, and β is the shape parameter representing a measure of the spread of the breakdown data.^26^

## Results and Discussion

### Structure of PU

**Figure 3:**
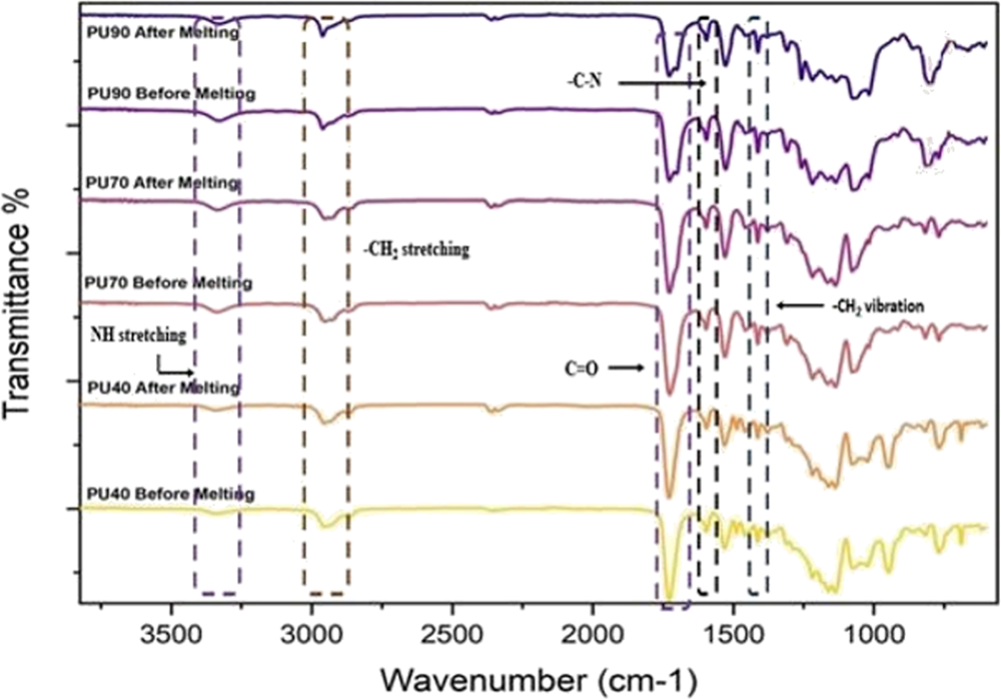
FTIR spectra of PU40, PU70, and PU90 before and after the melting process.

The FTIR spectra of all three types of PU before and after the melting process are shown in Figure 3. The spectral peak at 3336 cm^–1^ corresponds to NH stretching, while the peak at 2959 cm^–1^ is associated with −CH_2_ stretching. Further, hydrogen bonding between the secondary amine group (−NH) and the carbonyl group (C=O) is the intrinsic driving force for the phase separation of PUs, which is important for the self-healing performance.^27^ The hydrogen bonding interaction makes the carbonyl bond length elongated and results in the reduction of the stretching vibration frequency.^28^ Hence, mathematical deconvolution of the carbonyl stretch peaks around 1729 and 1704 cm^–1^ can be used to divide the free and hydrogen carbonyl.^29^ The higher intensities between the wavenumbers 1729 and 1704 cm^–1^ associated with the carbonyl stretch and H-bonded carbonyl in PU70 and PU90 samples indicate a higher concentration or abundance of hard segments in those samples. Conversely, the absence of these peaks in PU40 suggests a lower concentration of hard segments. Further, the peak at 1417 cm^–1^ is associated with −CH_2_ vibration.^21,22^ Moreover, the peaks at 1232, 1092, and 929 cm^–1^ can be attributed to the presence of aliphatic ether.^23^ The attributions of FTIR spectral peaks are tabulated in Table 1.

**Table 1:** Peaks Attributions of FTIR Spectra of PU

wavenumber (cm^–1^)	vibration
3336	N–H stretching H-bonded
2959	C–H stretching
1729	O=C free carbonyl
1704	HN–O=C H-bonded carbonyl
1525	C–H stretch, N–H bend
1417	–CH_2_ vibration
1307	C–N urethane
1232	asymmetric N–CO–O, C–H aliphatic skeleton
1092	C–O–C aliphatic ether
1017	symmetric N–CO–O
929	C–O–C stretch aliphatic ether
866	C–C skeleton vibration
775	C–C skeleton rocking

### Differential Scanning Calorimetry

**Figure 4:**
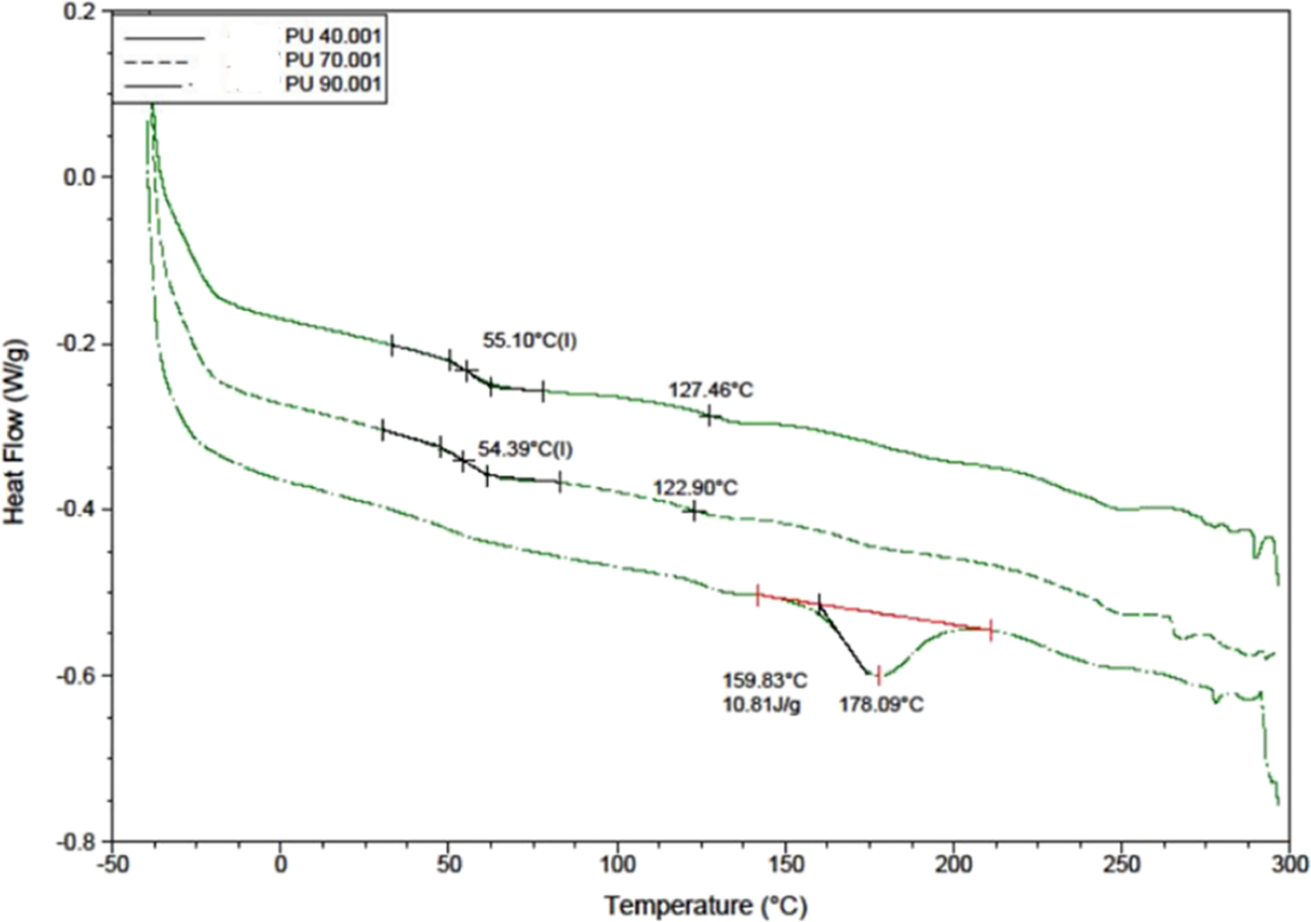
DSC measurements of PU40, PU70, and PU90.

The materials were subjected to DSC to explore the thermal characteristics of PU with varying levels of hardness. The DSC results are shown below in Figure 4. In PU40, two distinct glass transition temperatures were observed at 55.10 and 127.46 °C. The first temperature signifies the transition of the soft segment of PU40, while the latter represents the transition of the hard segment within PU40. This behavior was also noticed in PU70. A distinct process is observed in PU90 at 178 °C, which is associated with the melting of hard segments. A hint of a smaller version of a similar process can also be observed for PU40 and PU70.

### DC Conductivity Measurement

**Figure 5:**
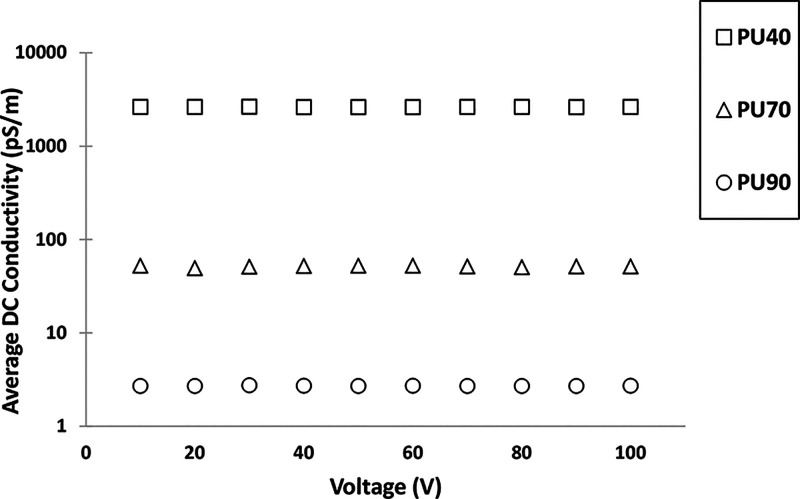
Average DC conductivities of PU40, PU70, and PU90 at different voltage levels.

Ten samples of each material were tested to analyze the DC conductivity. Figure 5 provides the individual DC conductivity values of ten samples of all the materials at various voltage levels. Further, Table 2 shows the average DC conductivity of each material with standard deviation in the measured conductivity in each PU variant. Further, the percentage variation in DC conductivity indicates minimal variability in the measurements. Given the small magnitude of the variation, it is reasonable to conclude that edge effects are negligible and unlikely to significantly impact the accuracy of the conductivity measurements.

**Table 2:** Average Conductivity of PU Variants

material	average DC conductivity	standard deviation
PU40	2.6293 nS/m	0.001 nS/m
PU70	51.4509 pS/m	0.097 pS/m
PU90	2.7103 pS/m	0.002 pS/m

### Dielectric Spectrum Analysis

**Figure 6:**
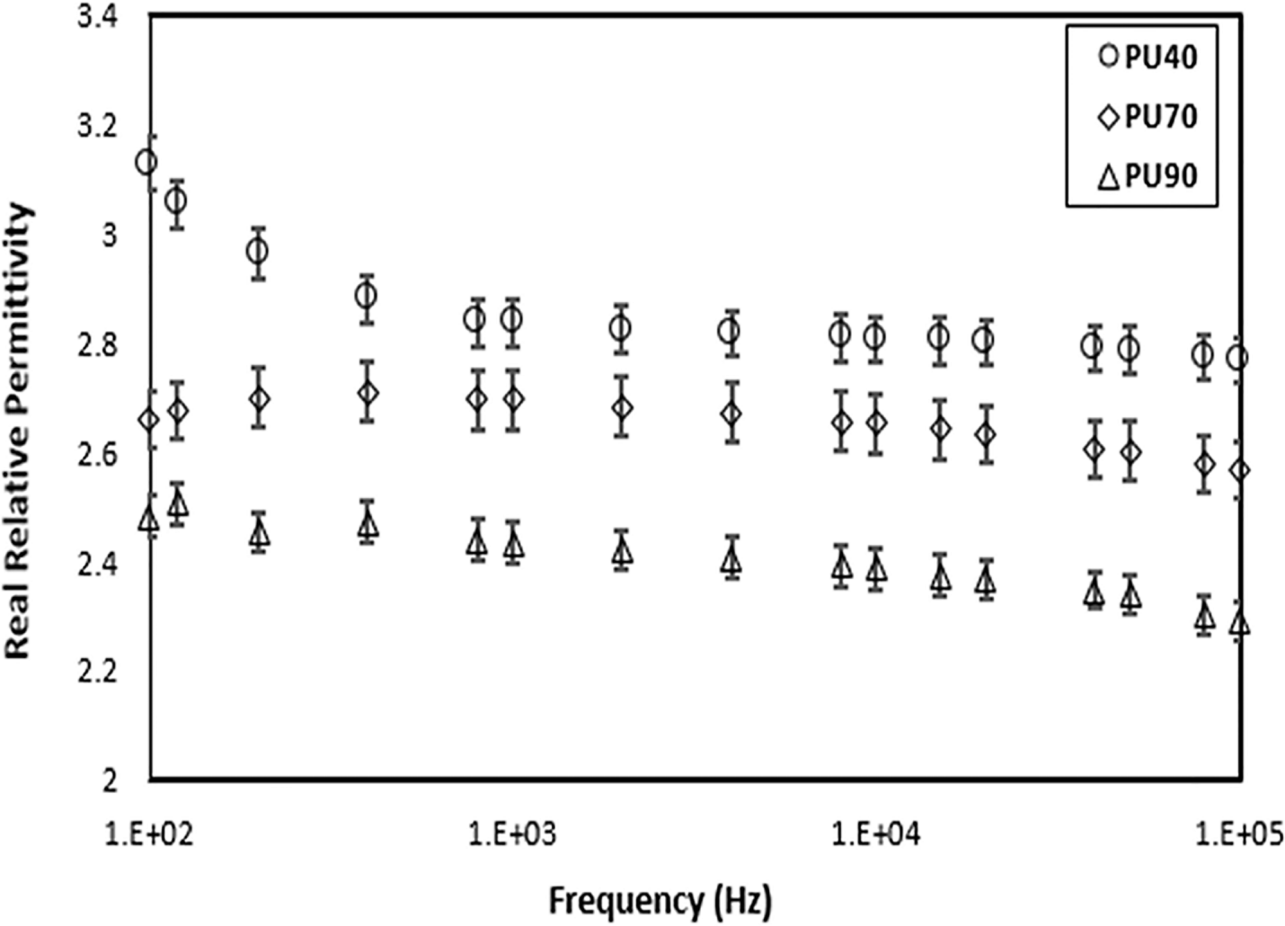
Real relative permittivity of PU40, PU70, and PU90 as a function of frequency.

**Figure 7:**
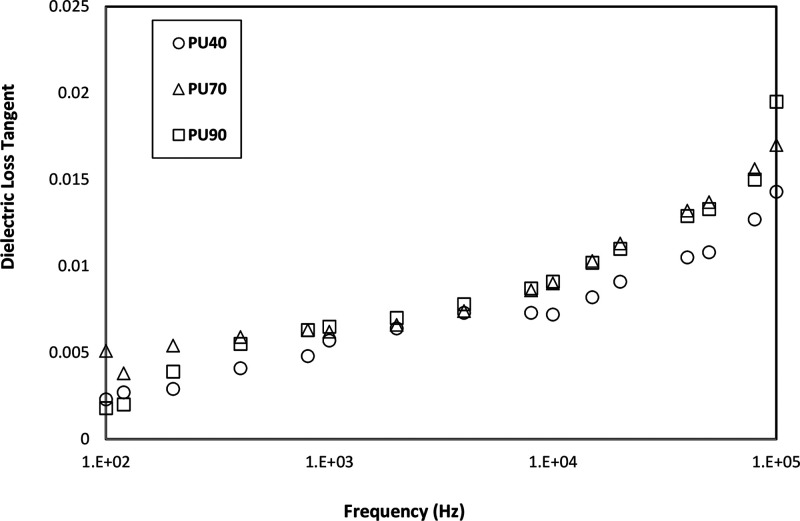
Dielectric loss tangent of PU40, PU70, and PU90 as a function of frequency.

Figures 6 and 7 depict the frequency-dependent behavior of the relative permittivity and dielectric loss tangent for each PU variant. Figure 6 illustrates that the change in real relative permittivity of the PU variants is high at lower frequencies. This behavior is more pronounced in softer PU and becomes less pronounced as the hardness of the PU increases. Similarly, a downward trend is observed in the real relative permittivity of the PU variants at frequencies above 1 kHz. Moreover, the real relative permittivity of the PU materials decreases as the hardness of the material increases. Furthermore, the dielectric loss tangent increases as the frequency is increased for all PU variants, as shown in Figure 7. Overall, the dielectric loss tangent of all materials exhibits a low value, indicating minimal energy loss within the PU samples.

## Dielectric Breakdown Properties of PU Variants

### AC Breakdown Strength

**Figure 8:**
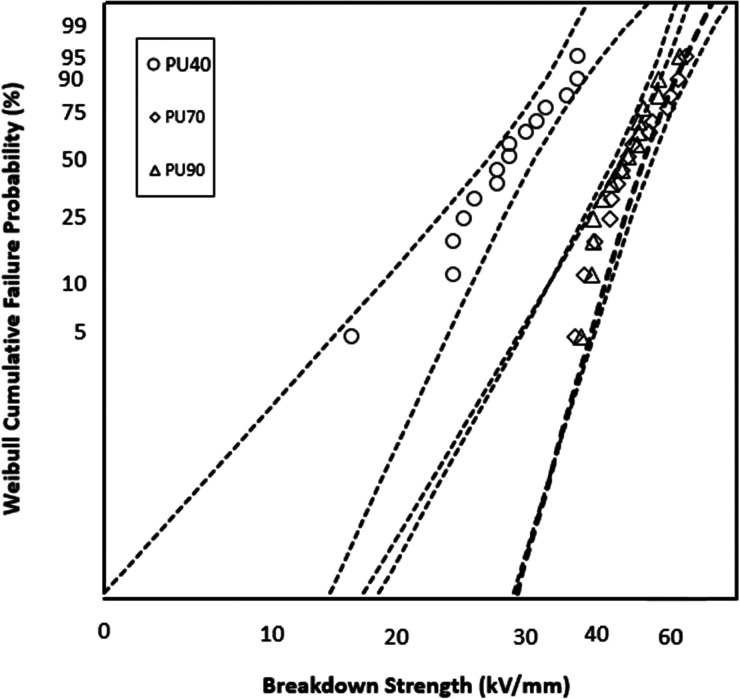
Weibull plots comparing the AC breakdown strengths of PU40, PU70, and PU90.

The variation in AC breakdown performance among the different PU variants was statistically analyzed. Figure 8 compares the AC breakdown strengths among the PU variants: PU40, PU70, and PU90. The AC breakdown strength based on 63% probability of failure for PU40 is 19 kV/mm, indicating a lower breakdown strength than PU70 and PU90. The AC breakdown strengths of PU70 and PU90 are found to be relatively close to each other. The corresponding Weibull data, detailed in Table 3, provide additional statistical analysis of the breakdown strength distribution for each PU variant.

**Table 3:** Weibull Parameters Comparing the AC Breakdown Strengths of PU40, PU70, and PU90

material	α (kV/mm)	β
PU40	19 ± 3	3 ± 1
PU70	36 ± 1	14 ± 6
PU90	39 ± 1	8 ± 3

### DC Breakdown Strength

**Figure 9:**
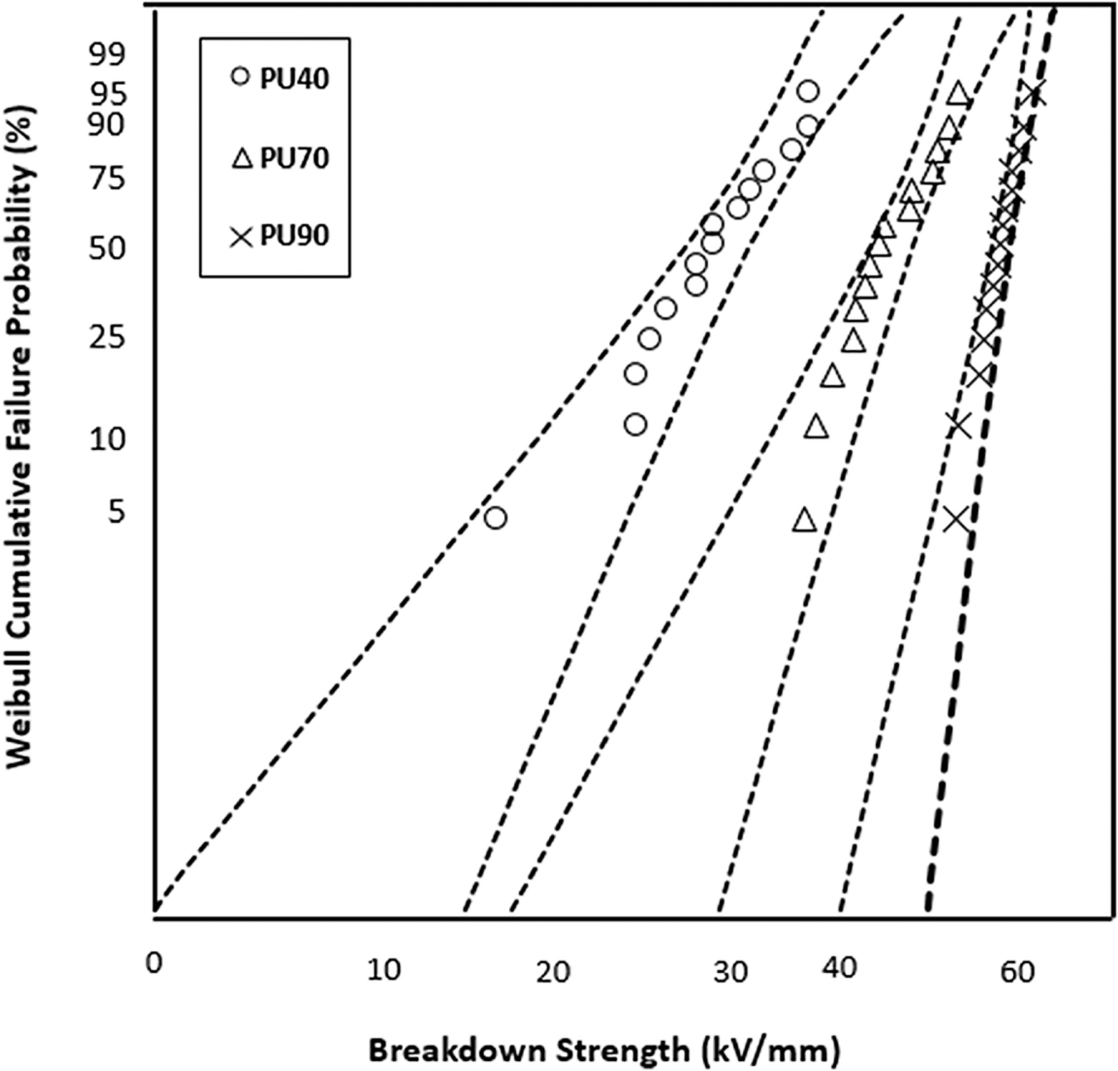
Weibull plots comparing the DC breakdown strengths of PU40, PU70, and PU90.

In Figure 9, a comparison is presented regarding the DC breakdown strength of the PU variants: PU40, PU70, and PU90. The corresponding values of DC breakdown strength for PU40, PU70, and PU90 were measured at 24, 41, and 56 kV/mm, respectively. Notably, as the hardness of the PU material increases, there is a noticeable trend of an increase in the DC breakdown strength. The Weibull data, tabulated in Table 4, further support these findings by statistical analysis of each PU variant’s breakdown strength distribution.

**Table 4:** Weibull Parameters Comparing the DC Breakdown Strength of Nylon, PP, PU 40, PU 70, and PU90

material	α (kV/mm)	β
PU40	24 ± 3	5 ± 2
PU70	41 ± 3	7 ± 3
PU90	56 ± 2	18 ± 7

## Discussion

In Figure 3, based on the FTIR spectral analysis of all the materials, it can be seen that there was no shift in spectral peaks for all PU variants before and after melting, which implies that the melting process did not induce any chemical change in the material. Further, a shift in the magnitude of the spectral peaks of PU90 was observed after the melting process. The decrease in magnitude of the spectral peaks in PU90 after melting indicates the changes in physical properties of the material due to the melting process which can be either structural or morphological or both.^30^ The authors believe that apparently this physical change affected the AC breakdown characteristics of PU90 more than DC, likely attributed to the shift in polarity associated with AC, as shown in Figures 8 and 9. In Figure 5, the DC conductivity analysis showcases the behavior of PU across different voltage levels. It was observed that the DC conductivity of all PU variants was independent of the applied electric field. Moreover, a noticeable trend emerges where the DC conductivity of the PU decreases as the hardness of the PU increases. Further, the DC conductivity of PU40 is 50 times greater than that of PU70 and the conductivity of PU70 is 25 times greater than the conductivity of PU90, as shown in Table 1. The decrease in DC conductivity of PU with the increase in hardness of the PU is attributed to the internal structure of the PU. As the hardness of PU is regulated by adjusting the proportion of hard segments; therefore, the increase in hardness of PU indicates the presence of more hard segments and significant interactions between them. These effectively act as cross-links and reduce the mobility of the polymer chains, therefore reducing the mobility of charge carriers ultimately resulting in lower DC conductivity. The observed relationship between material hardness and DC conductivity highlights the importance of material selection and understanding the electrical behavior of PU variants in high-voltage applications.

Further, the graph in Figure 6 depicts the relationship between frequency and real relative permittivity for all PU variants. Analyzing the dielectric spectra, it can be seen that the real relative permittivity of the PU40 material is increasing at lower frequencies unlike PU70 and PU90. The increase in the measured real relative permittivity at low frequencies has been attributed to electrode polarization effects associated with conductivity. In liquid systems, an expression in the form of eq 2
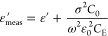
2has been used to relate the measured real relative permittivity (ε_meas_^′^) to the physical permittivity ε′, where σ is the conductivity, while *C*_0_ is the geometrical capacitance and *C*_E_ is the capacitance associated with the interface between the electrode and the liquid.^31^ The situation in solid dielectrics is more complex, but similar behaviors are observed with an increase in the measured real relative permittivity occurring at low frequencies when conductivity allows the movement of ionic charge within the polymers.^32^

Overall, the real relative permittivity of the PU variants decreases as the frequency is increased. This behavior can be attributed to the intrinsic properties of the material and its response to the applied electric field. The material exhibits a higher real relative permittivity at lower frequencies, indicating a more robust polarization response to the electric field. However, as the frequency increases, the material’s ability to align and reorient its electric dipoles becomes less efficient, resulting in a decrease in the real relative permittivity. Additionally, the dielectric loss tangent increases with frequency for all PU variants which indicates the impact of the relaxation process of materials domains at higher frequencies. it is worth noting that all the materials exhibit very low dielectric loss tangent values. This low value implies minimal energy loss within the materials upon being subjected to the electric field, indicating their potential for electrical insulation applications.

An interesting observation can be made regarding the AC and DC dielectric breakdown characteristics. The breakdown strength increases as the PU’s hardness increases. This phenomenon suggests that PUs with higher hardness levels can withstand electrical breakdown and display higher strength. The underlying reasons behind this correlation between the hardness and breakdown strength can be attributed to several factors. PUs with higher hardness levels possess more hard segments in their composition. This higher concentration of hard segments reduces the mobility of charge carriers and results in a higher dielectric breakdown strength.

The trend becomes even more pronounced upon examination of the DC breakdown strength. Interestingly, the DC dielectric breakdown strength data for PU70 and PU90 exhibit distinct spacings, unlike the overlapping Weibull plots observed in the AC breakdown. The authors made the following assumptions to explain this behavior. The difference in DC and AC breakdown strength of PU90 compared to that of PU70, with higher values observed under positive DC compared to lower values under AC for PU90, can be attributed to the unidirectional nature of the DC stress. Under positive DC conditions, the electrical stress is consistently applied in one direction, leading to specific charge accumulation and polarization effects within the material. This unidirectional stress may result in a higher breakdown strength as compared with the alternating nature of AC, where the reversal of the electric field can influence charge distribution differently. Additionally, the frequency dependence of AC breakdown, coupled with the potential for dielectric loss and heating effects, may contribute to the observed lower breakdown strength under AC conditions. Similar observations regarding the AC and DC breakdown strength of thin polymeric films have already been observed and reported.^26,33^ Further, the FTIR results revealed that the melting process of PU90 affected its physical characteristics, which influenced the AC characteristics of PU90 more than the DC characteristics. Overall, the breakdown strength of PU variants was low as compared to polyethylene and XLPE,^26,34^ but it showed that increased hardness corresponds to higher dielectric strength. This observation prompts further investigation into the synthesis process, specifically focusing on the ratio of hard and soft segments in the material.

## Conclusions

The dielectric breakdown characteristics of PU materials exhibit a consistent trend in both AC and DC scenarios. The experimental results demonstrate that as the hardness of PU increases, the dielectric breakdown strength also increases apart from AC breakdown tests for PU70 and PU90 where the dielectric breakdown strengths of these two materials were close to each other. This correlation between hardness and breakdown strength can be attributed to a higher proportion of hard segments which reduces the mobility of charge carriers, which contribute to improved dielectric breakdown strength. The self-healing capability of PU may decrease as the material’s hardness increases, whereas the dielectric strength of PU demonstrates an increase with hardness. Consequently, further investigations are necessary to explore the trade-off between the dielectric characteristics and self-healing capabilities of PU. Understanding this trade-off is crucial for potential applications of PU as an insulation material in high-voltage systems.
